# Home-Based Speech Perception Monitoring for Clinical Use With Cochlear Implant Users

**DOI:** 10.3389/fnins.2021.773427

**Published:** 2021-11-30

**Authors:** Astrid van Wieringen, Sara Magits, Tom Francart, Jan Wouters

**Affiliations:** Experimental ORL, Department of Neurosciences, KU Leuven, Leuven, Belgium

**Keywords:** speech understanding in noise, digits in noise, phoneme identification in quiet, CI users, home testing

## Abstract

Speech-perception testing is essential for monitoring outcomes with a hearing aid or cochlear implant (CI). However, clinical care is time-consuming and often challenging with an increasing number of clients. A potential approach to alleviating some clinical care and possibly making room for other outcome measures is to employ technologies that assess performance in the home environment. In this study, we investigate 3 different speech perception indices in the same 40 CI users: phoneme identification (vowels and consonants), digits in noise (DiN) and sentence recognition in noise (SiN). The first two tasks were implemented on a tablet and performed multiple times by each client in their home environment, while the sentence task was administered at the clinic. Speech perception outcomes in the same forty CI users showed that DiN assessed at home can serve as an alternative to SiN assessed at the clinic. DiN scores are in line with the SiN ones by 3–4 dB improvement and are useful to monitor performance at regular intervals and to detect changes in auditory performance. Phoneme identification in quiet also explains a significant part of speech perception in noise, and provides additional information on the detectability and discriminability of speech cues. The added benefit of the phoneme identification task, which also proved to be easy to administer at home, is the information transmission analysis in addition to the summary score. Performance changes for the different indices can be interpreted by comparing against measurement error and help to target personalized rehabilitation. Altogether, home-based speech testing is reliable and proves powerful to complement care in the clinic for CI users.

## Introduction

Speech perception assessment is a cornerstone of audiological rehabilitation (Boothroyd, 1994). It is usually assessed in the clinic with meaningful words and sentences in quiet and (sometimes) in noise. These scores reflect large variability in performance for persons with hearing aids (HA) and cochlear implants (CI), especially in noise (e.g., Gifford et al., 2008, 2015; Zeitler et al., 2008; Meister et al., 2015; Ricketts et al., 2019), due to differences in patient demographics, as well as technical, linguistic, and cognitive factors (Rählmann et al., 2018; James et al., 2019; de Graaff et al., 2020; Zhao et al., 2020). The multidisciplinary nature of audiological rehabilitation requires a wide range of performance measures to capture bottom-up and top-down neurocognitive skills (Moberly et al., 2016; Rählmann et al., 2018; Skidmore et al., 2020; Tamati et al., 2020; Völter et al., 2020; Zhan et al., 2020; Biever et al., 2021; Lundberg et al., 2021). However, clinical time is scarce. Demand for care has increased over the past years due to the expansion of candidacy criteria for cochlear implantation, advancements in technology, and improved surgical techniques (van der Straaten et al., 2020; Perkins et al., 2021). A potential approach to alleviating some of the work on clinical care and possibly making room for other outcome measures is to employ technologies that assess performance in the home environment. An increasing number of people are using their smartphones or tablets for healthcare assessment, and home-based testing could be used to monitor potential changes in hearing performance and provide guidance for audiological rehabilitation. Such an approach may be good for the clinic (reduced workload/more testing) and enhance the user’s self-efficacy.

Over the past decade, different audiological service deliveries via telepractice have been explored (Swanepoel and Hall, 2010; Muñoz et al., 2021). Several applications for hearing screening have demonstrated the feasibility and reliability of telehealth (Smits et al., 2013; Louw et al., 2017). For experienced HA users, face-to-face and remote programming of hearing aids give similar speech perception results (Venail et al., 2021). Also, CI programming levels are similar when done remotely compared to the face-to-face method in the clinic, not only with adults (Ramos et al., 2009; Wesarg et al., 2010; Hughes et al., 2012; Eikelboom et al., 2014) but also with children using visual reinforcement audiometry (Hughes et al., 2018). Additionally, speech recognition of CI users can be assessed at home (de Graaff et al., 2018, 2019), although presentation mode requires some attention. With direct-connect from the computer to the CI processor, different physiological and basic perceptual measures yielded similar scores whether assessed in person or remotely (Goehring et al., 2012; Hughes et al., 2012). However, speech perception scores of CI users were significantly poorer in an office/conference room simulating remote testing than in the in-person condition in the sound booth at the clinic, presumably because of the higher background noise level and longer reverberation times at the remote sites. To overcome the adverse effects of background noise and reverberation, speech sounds can be delivered via direct audio input (DAI), bypassing the microphones. While testing with DAI has proved to be a valid alternative to standard sound-booth testing (de Graaff et al., 2016, 2019; Cullington and Aidi, 2017; Sevier et al., 2019), wireless streaming from a device to the sound processor has also become possible and can be used for testing in the home environment.

Using remote tools may also lead to increased confidence to manage one’s hearing and identify problems quicker instead of waiting for a scheduled appointment at the clinic. A randomized control trial using a well-validated generic measure of patient activation showed that CI users who received remote care for device adjustment and assessment demonstrated greater user activation after 6 months than those who received the clinic-based care pathway (Cullington et al., 2018). A custom-made satisfaction questionnaire revealed that patients and clinicians were generally positive about remote care tools and wanted to continue. They liked the idea that tests can be used any time, that they receive instant feedback on progress, and that less staff is needed. These findings related to audiological rehabilitation align with a systematic review analysis and meta-analysis showing that self-management support interventions can reduce health service utilization without compromising patient health outcomes (Panagioti et al., 2014).

Not all outcome measures are suitable for remote self-testing. In the clinic, speech understanding is usually assessed with an open-set response format. The client responds verbally to the presented word or sentence, and the clinician notes down the responses. Home-based testing requires a closed-set response format, where the client chooses from a pre-defined set of alternatives unless auto-correction is applied with open-set testing (e.g., Francart et al., 2009). Another prerequisite for home-based testing is that the materials can be used repeatedly. Meaningful words and sentences cannot be used repeatedly unless an infinitive number of alternatives can be generated, such as with the Matrix sentences (Kollmeier et al., 2015) or the Coordinate Response Measure (Bolia et al., 2000). Digits and phonemes can be used repeatedly.

The digit triplet test also called the digits in noise test (DiN), is increasingly used for hearing assessment. It was initially developed for hearing screening (Smits et al., 2013; for a review, we refer to Van den Borre et al., 2021), but with persons with a cochlear implant, it is also used as an alternative for the sentence in noise (SiN) task (Kaandorp et al., 2016; Cullington and Aidi, 2017; Zhang et al., 2019). Using an adaptive procedure, the speech reception threshold is determined for digits presented in speech-weighted noise. Even persons with limited language ability are familiar with digits and can use a keypad. Long before this paradigm was developed, it was clear that an extensive range of hearing abilities can be mapped with numbers (van Wieringen and Wouters, 2008). The DiN paradigm can be used repeatedly since learning of the content is less likely to occur.

Phoneme identification, or the nonsense syllable test, is also assessed with an n-alternative closed-set response format. The summary scores (percentage correct) reflect how well a listener perceives the spectral and temporal properties of vowels and consonants (e.g., Gordon-Salant, 1985; Dorman et al., 1990; Tyler and Moore, 1992; van Wieringen and Wouters, 1999; Välimaa et al., 2002a,b; Munson et al., 2003; Nie et al., 2006; Shannon et al., 2011; Rødvik et al., 2018). Phoneme identification is not often assessed in the clinic, although responses are very insightful, as they can yield both a summary score and detailed analysis of confused speech features by means of information transmission analyses (Miller and Nicely, 1955). Phonemes are characterized by distinctive acoustic features that produce differences in voicing, manner, place of articulation, etc., Per phoneme, the transmission of different speech features is determined. The relative information transmitted is the ratio of the transmitted information calculated from the confusion matrix to the maximal possible information transferred by the stimuli and features under test. The more phonemes share distinctive features, the more likely they are confused perceptually (Miller and Nicely, 1955). The results of the information transmission analysis can guide the rehabilitation process (e.g., optimize the fitting of the device). Nonsense syllable tests also have the advantage that learning effects in multiple experiments with the same stimuli are minimal compared with tests using real-word stimuli (Dubno and Dirks, 1982).

In summary, clinical care is time-consuming and often challenging with an increasing number of clients. Speech-perception testing is essential for monitoring outcomes with a HA or CI and should encompass various measures to gain insight into variability in performance. Some of these could be done at home to complement assessment in the clinic. The study aimed to investigate performance on three different speech perception tasks, i.e., sentence identification in noise (SiN), digits in noise (DiN), and phoneme identification in quiet, in the same CI recipients during 16 weeks. We expect the digit scores to be associated with the sentence scores, and we anticipate that the vowel and consonant errors will provide additional insight into individual performance patterns. Additionally, we investigate the reliability of these indices in the home-based setting and potential differences between response scores determined at the beginning and at the end of the trial.

## Methodology

### Participants, Outcome Measures and Procedure

Forty CI users, 26 with Cochlear device, 14 with AB device, performed the phoneme and DiN tasks at home. Their median age was 64.3 years [IQR 10.4, min 28 yrs, max 75 yrs], median experience with their CI 2.1 years [IQR 4.2 yrs, range 0.1–15.9 yrs]. Thirty-six out of forty CI users had progressive hearing loss. Twenty-seven participants wore a hearing aid contralaterally (CI-HA), eight persons had one CI, three persons bilateral CIs, and two persons 1 CI and residual hearing. The participants’ average pure tone average (PTA4, average of 500, 1,000, 2,000, 4,000 Hz), determined in free field at the clinic with their CI only, was 26.4 dB HL (SD 5.3). All participants presented with a postlingually acquired profound hearing impairment, and they communicated through spoken language in their daily life. The median period of education was 12.5 years [IQR 3.3].

These participants participated in a more extensive study dealing with the efficacy of a personalized listening training program LUISTER compared to a non-personalized one (Magits et al., under revision). In that study, participants were asked to practice segmental and suprasegmental speech tasks five times per week for 15 to 20 min on a tablet at home. The efficacy of the two training tasks was based on the SiN scores (pre- versus post-training) assessed at the clinic. Once a week, before practicing with a training program, the participants were asked to complete a DiN test twice and either a vowel or a consonant phoneme identification task (in quiet) at home. At home, the stimuli were streamed via Bluetooth and a streaming device to one CI. The participant chose which CI if they had two. Speech understanding in noise (SiN, pre-and post-training) was assessed at the clinic, via streaming. Three conditions were tested: (1) SiN presented via streaming to one CI (same as DiN and phoneme in quiet, “SiN streaming”), (2) in sound field to the CI only (“SiN CI-SF”), and (3) in sound field as in daily life (with CI and HA if applicable, “SiN daily settings”). The same CI devices were used at home and at the clinic. Logged data were automatically transferred to a repository hosted on the server of the research group via a restricted one-way communication from tablet to server.

Participants provided written informed consent, and the Ethics Committee approved the study of the University Hospitals Leuven (approval no. B322201731501). Participants were paid for the testing sessions but not compensated for the practicing sessions at home. The study protocol is registered on ClinicalTrials.gov (I.D. = NCT04063748).

### Outcome Measures

#### Speech Understanding in Noise (SiN)

Sentence understanding in stationary speech-weighted noise (SiN) was assessed with the LIST speech materials (van Wieringen and Wouters, 2008). An adaptive method was used to determine the speech reception threshold (SRT), the signal-to-noise ratio at which 50% of the sentences are repeated correctly. Each sentence contains two to three keywords. The level of the sentences was held fixed at 65 dB SPL, the level of the noise was varied. The level of the noise for the first sentence varied until all keywords were repeated correctly. For each subsequent sentence, the level of the noise was increased or decreased in steps of 2 dB until ten sentences had been presented (Plomp and Mimpen, 1979). The SRT was the average of the last five presented signal-to-noise ratios and the signal-to-noise ratio of the imaginary 11th sentence, with lower SRT values indicating better performance.

Participants completed two lists for each of the three conditions before and at the end of the 16-week trial. A third list was completed if the two lists differed by more than 2 dB, and the average was taken. In the sound field room at the clinic speech sounds were played using APEX (Francart et al., 2017) from a tablet via a streaming device to the CI or a computer via an external sound card to the loudspeaker at 65 dB SPL. The median duration for SiN testing ranges from 2.2 min [0.5 min] to 2.4 min [0.7 min] per list of 10 sentences, hence 6–8 min in total.

#### Digits in Noise (DiN)

Participants identified 17 digit triplets in stationary speech-weighted noise on the touch screen of the tablet. The development and validation of the Flemish DiN (female speaker) are described by Jansen et al. (2013). The level of the speech was fixed at 65 dB A, and the first triplet was presented at + 4 dB signal-to-noise ratio. An adaptive procedure using triplet and digit scoring and an adaptive step size converged to a threshold in noise (Denys et al., 2019). One DiN trial takes about 2.3 min [0.4 min].

#### Phoneme Identification in Quiet

Both vowel and consonant identification in quiet were assessed separately. The vowel identification task consisted of 10 Dutch/Flemish vowels presented in p-t context: /oe, oo, i, I, o, u, e, ee, aa, a/. The consonant identification test consisted of 12 consonants presented in/a/context: /p, t, b, d, m, n, s, f, ch, z, v, w/. Stimuli were produced by a female speaker (van Wieringen and Wouters, 1999). Each phoneme was routed ten times from the tablet to the streaming device in random order (*n* = 100 for vowel, *n* = 120 for consonant). Testing was self-paced. No training nor feedback was provided. Vowel identification (100 items) takes 6.0 min [2.2] and consonant identification (120 items] takes 9.4 min [2.8 min].

Responses were cast into stimulus-response confusion matrices. Information transmission (Miller and Nicely, 1955) was determined of three speech features for the Dutch vowels: Duration, First formant frequency (F1), and Second formant frequency (F2). Classification of the vowels into these categories is the same as documented in van Wieringen and Wouters (1999, Table 3). Seven features distinguish consonants: presence/absence of voicing (voicing), perception of release burst (plos), perception of relatively high or low amplitude envelope (envel), place of articulation (place), perception of frication (fric), manner of articulation (manner), and perception of nasal cues (nasal). The classification of the consonants into these categories follows van Wieringen and Wouters (1999, Table 5).

### Procedure

#### Tablet and Calibration

Testing was done with a 7.0″ Samsung Galaxy Tab A tablet and a streaming device, the phone clip or minimic for the Cochlear device (*n* = 26) and compilots for the AB device (*n* = 14). The output level for the speech tasks was calibrated with a personal audio cable, and the overall intensity level was set to 65 dBA. During the initial visit at the clinic participants were shown how to connect their streaming device and to run the tasks. A blue light indicated that the streaming device was connected. Participants also received manuals with clear instructions or could contact the clinician via email if needed. They were allowed to adjust the volume settings of their streaming devices but nobody reported having done this. At the end of the 16 weeks participants were asked to rate the usability of the tablet using the System Usability Scale (from 0 to 100), developed by Brooke (1996). The average SUS score was 90.5 (SD 10.4), the median is 95 (IQR 5).

#### Number of Trials

All participants performed the DiN test twice sequentially and completed either a vowel identification task or a consonant identification task each week during the 16 weeks. This resulted in 1269 DiN trials, 307 vowel identification trials, and 326 consonant identification trials. The average number of trials per person was 31.7 (SD 4.1) for the DiN 7.7 (SD 1.0) for vowel identification and 8.2 (SD 1.2) for consonant identification, respectively. Since 2 (out of 40) participants performed the vowel and consonants tasks only five times, the averaged values of DiN and phoneme identification are based on the last five trials (=weeks) per participant. SiN is based on one value (average of 2–3 lists of sentences), determined in the first week and one value determined in the last week.

### Statistics

Statistical analyses were performed using IBM SPSS Statistics for Windows version 27 (2020). Data were tested for normality and homogeneity of variance. The Shapiro-Wilk showed that the DiN data distribution did not significantly differ from normal, *W*(40) = 0.946, *p* = 0.057, but the SiN data did *W*(40) = 0.907, *p* = 0.003. Vowel identification scores were normally distributed: *W*(40) = 0.978, *p* = 0.628, as well as consonant identification scores: *W*(40) = 0.984, *p* = 0.845. The pure tone average (PTA) data were also normally distributed, *W*(40) = 0.967, *p* = 0.296, but not “years of CI use,” *W*(40) = 0.836, *p* < 0.001. Since the SiN data were not normally distributed we opted for median and interquartile ranges when presenting SiN with other performance measures. The non-parametric Spearman’s Rho was used to determine the strength of an association between SiN and other variables, while Pearson correlation (r) was used for the normally distributed performance measures. Linear regression analyses were performed to study the relationship between different performance measures and to determine how much the different predictors explain the response. Potential differences in performance between the start and end of the 16-week trial were analyzed with the non-parametric Friedman test of differences among repeated measures, followed by a Wilcoxon signed rank test for paired comparisons.

## Results

### SiN and DiN

**FIGURE 1:**
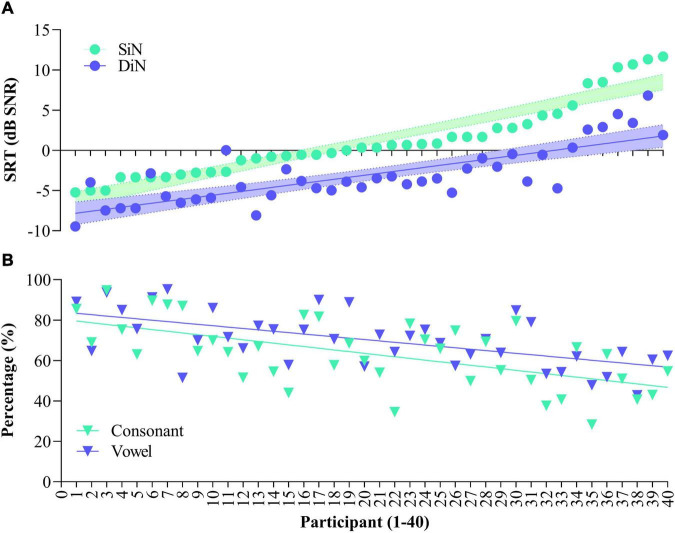
Speech reception thresholds for Sentence in noise (SiN) and Digits in noise (DiN) for the 40 participants **(A)** and concomitant vowel and consonant scores [percentage correct, **(B)**]. Data are ranked according to SiN. DiN and phoneme recognition data are based on the average of the last 5 trials.

Figure 1 illustrates SiN (streaming) and DiN (A) and percentage vowel and consonant identification in quiet (B) for each of the 40 participants separately. Participants are ranked according to increasing (poorer) SiN scores determined at the end of the 16-week trial. These scores range from –5.2 to + 11.6 dB SNR. Generally, DiN scores are in line with the SiN ones by 3–4 dB improvement. The median SRT of the last five trials for DiN is –3.8 dB SNR [IQR, 5.0), and for SiN streamed to the device –0.3 dB SNR [IQR, 4.5). The difference between the DiN and the SiN in this study, about 3.5 dB, is also in line with the difference between the norm values of the SiN for normal hearing young persons (−7.8 dB SNR, van Wieringen and Wouters, 2008) and the norm values of the DiN (−11.7 dB SNR, Jansen et al., 2014).

Spearman’s rho indicates a statistically significant relationship between the SRTs of SiN and DiN (*rs* [40] = 0.767, *p* < 0.001). Linear regression analyses showed that DiN significantly predicts SiN, thereby explaining 74% of the variance, *F*(1,38) = 621.34, *p* < 0.0001. The model for SiN is *y* = 4.52 + (1.098 *score) with a narrow 95% confidence interval to predict SiN from the DiN score [3.5–5.4].

### Phoneme Identification in Quiet

The bottom panel (Figure 1B) illustrates phoneme identification in quiet for each of the participants. All participants performed well above chance (10% for vowels and 8.3% for consonants), but a wide range of performance is observed. Median vowel identification is 70.0% [IQR 17.8], median consonant identification is 64.4% [IQR 24.1]. Vowel and consonant perception in quiet are highly correlated [*r*(40) = 0.678, *p* < 0.001], the difference between the two measures is in the same order of magnitude for most participants.

Spearman’s rho indicated a significant negative relationship between SiN assessed at the clinic and vowel identification in quiet assessed at home (*rs* [40] = −0.611, *p* < 0.001), and a significant negative relationship between SiN and consonant identification in quiet (*rs* [40] = −0.587, *p* < 0.001). In other words, the more negative (better) sentence identification in noise, the higher the vowel and consonant recognition in quiet. Vowel and consonant recognition significantly predict SiN, with the linear regression model explaining 41% of the variance (*p* < 0.001). Semi partial correlations, which explain the unique contribution of each predictor variable, are 28% for vowel identification, *p* = 0.031, and 26% for consonant identification, *p* = 0.043.

As with SiN, a significant negative relationship was observed between DiN and vowel identification: *r* (40) = −0.537, *p* < 0.001, and between DiN and consonant identification: *r* (40) = −0.520, *p* = 0.001.

Vowel and consonant identification also significantly predict DiN, albeit somewhat less than SiN: the model explains 30% variance (*p* < 0.001). Semi partial correlations show that vowels predict 25% and consonants predict 21% of DiN.

### Perception of Vowel Features

**FIGURE 2:**
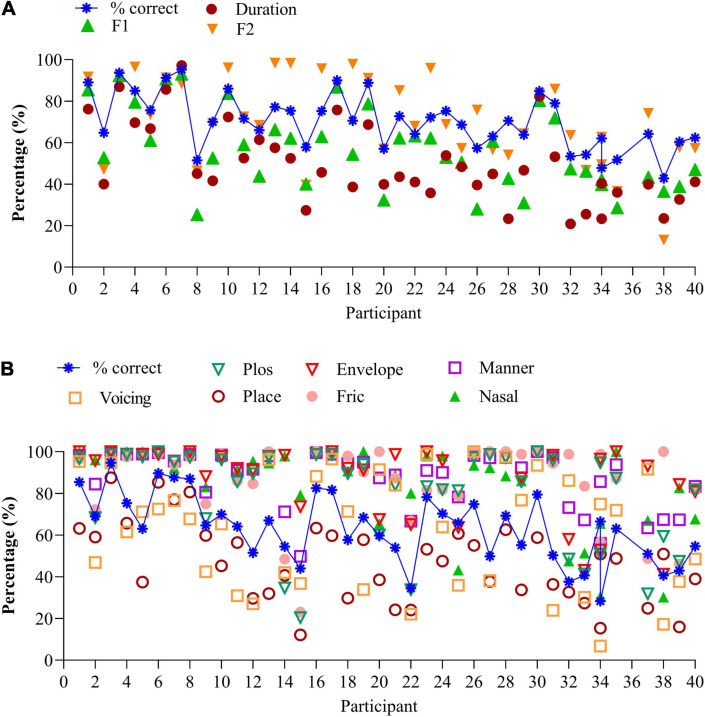
**(A)** Vowel identification for the 40 participants, together with the speech features. Data are ranked according to increasing (poorer) SiN scores. **(B)** Consonant identification for the 40 participants, together with the speech features. Data are ranked according to increasing (poorer) SiN scores.

Figure 2A illustrates the average percentages for each of the three vowel features for each 40 participants, together with the percentage correct score (blue stars). Participants are ranked according to increasing (poorer) SiN, as in Figure 1. Averaged over participants, “duration” is 50.4% (SD (19.0%), “F1” is 57.1% (SD 19%) and “F2” is 72.5% (SD 21%). F2 predicts 65% of the variance for vowel identification and was significant *F*(1,305) = 574.0, *p* < 0.0001. The linear regression analysis was performed on the individual data. Due to multicollinearity, the features “duration” and “F1” were dropped from the model (*r* > 0.7).

Information transmission analyses show that CI users with similar percent correct recognition scores can make different errors. Compare, for instance, vowel recognition of participants 15, 20, and 26 in Figure 2A. While percentage correct scores are similar (57%), the distributions of errors are different: participant 26 perceives the high-frequency spectral information much better (F2 cue, 76%) than participant 15 (40%) or participant 20 (57%). Likewise, participant 30 discriminates long and short vowels much better (80%) than participant 31 (53%) despite similar percentage correct scores (80%). Compare the data of participants 2 & 25, 21 & 23, and others who have similar percentage correct scores but perceive the different speech features differently.

### Perception of Consonant Features

Figure 2B illustrates the average percentages of the seven features per participant, together with the percentage correct scores ranked from low to high (blue crosses). Again, the order of participants is according to Figure 1 (SiN). Perception of voicing (AVG 61.6%, SD 27.1%), and place of articulation (AVG 47.0%, SD 19.1%) remain difficult, but the perception of plosives (AVG 79.2%, SD 23.4%), the coding of temporal envelope cues (envelop, AVG 88.2%, SD 16.6%), manner of articulation (AVG = 87.1%, SD 13.8%), fricatives 86.2% (SD 18.6%), and nasals (AVG 84.8%, SD 20%) are generally good. As with vowel identification, the feature transmission analyses of the consonant confusions provide additional information on differential performance.

The perception of the seven features can vary widely for a similar percentage correct score: compare, for instance, the data of participants 14 and 21 who both have similar recognition scores (54%). However, participant 21 mainly has difficulty perceiving place of articulation and perceives the other cues very well (>80%). In contrast, participant 14 has difficulty perceiving the correct place of articulation, perception of the burst, and frication (all below 50%). Compare also data of participants 6, 7, & 8, 9 & 11 to name a few. These participants yield the same percentage correct score, yet a different distribution of errors. Analysis of the errors can guide the mapping of the device and the rehabilitation process.

Of the seven consonantal speech features, “voicing” and “frication” significantly predict 53% of the variance of consonant identification in quiet, *F*(2,323) = 186.1, *p* < 0.0001 (*n* = 326). Both features contribute uniquely to predicting consonant recognition in quiet (*r* = 0.433 for “frication” and *r* = 0.3 for “voicing.” Due to multicollinearity (*r* > 0.7) the features “plos,” or perception of release burst (*r* = 0.77), envelope (*r* = 0.72), place of articulation (*r* = 0.85), manner of articulation (*r* = 0.8), and nasal (*r* = 0.72) were removed from the model.

### Longitudinal Analyses and Measurement Error

**FIGURE 3:**
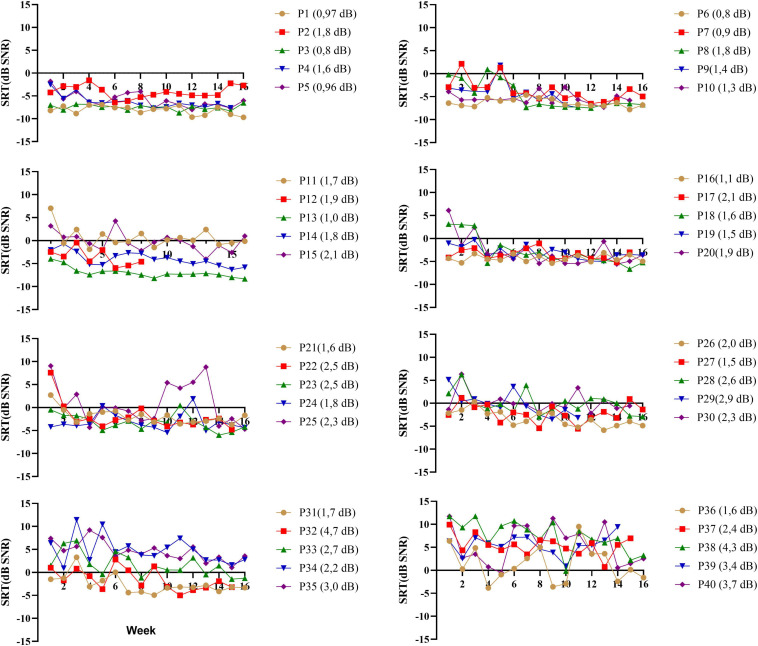
DiN thresholds as a function of week (*n* = 16) for each participant (p*) separately. Each data point is based on two estimates taken consecutively. Individual measurement errors are indicated next to the participant number and range from 0.8 dB (participant 3) to 4.7 dB (participant 32). Participants (P1-P40) are ranked according to increasing SiN.

A primary reason to use the DiN test is the limited content learning and thus the possibility to use the paradigm repeatedly (Smits et al., 2013). During the 16-week training trial (Magits et al., under revision), participants performed the DiN at home each week. Figure 3 illustrates the speech reception thresholds (SRTs) as a function of time for each participant separately (ranked in the same order as in previous figures). Each data point is the average of 2 SRTs administered consecutively at the beginning of each week. For the sake of clarity each figure illustrates the data of 5 participants. Most participants yield low SRTs that vary minimally with time, especially those with very good SiN scores (participants 1–10). Test-retest reliability was determined for each participant by taking the standard deviations of the differences between the two consecutive scores, divided by square 2. This procedure outbalances a procedural learning effect (Smits and Houtgast, 2005). Averaged over all participants, the measurement error is 2.0 dB (SD 0.9). Individual measurement errors are indicated next to the participant number in Figure 3 and range from 0.8 dB (participant 3) to 4.7 dB (participant 32). Generally, the values are larger for the participants with poorer DiN (and SiN), cf participants 30–40. These higher and more variable measurement errors were not related to the age of the participants (*r* (40) = −0.595, *p* = 0.09).

### Changes in Phoneme Identification in Quiet With Time

**FIGURE 4:**
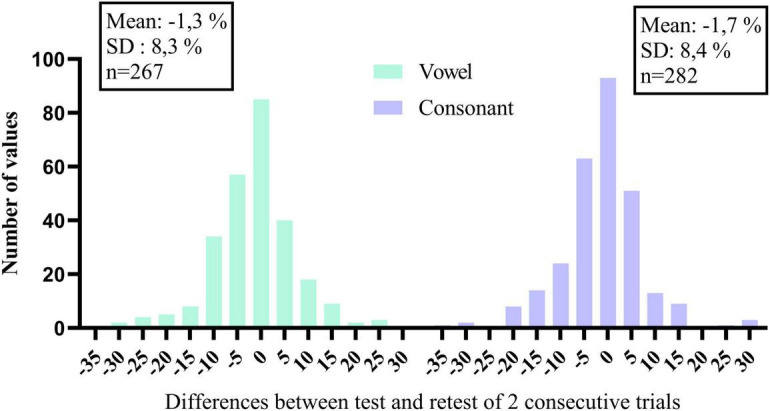
Histograms of the differences in percentage correct of consecutive trials, for vowels and consonants separately.

Closed-set phoneme identification in quiet also offers the possibility to monitor changes with time. Recall that the vowel and consonant tasks were performed every other week during the 16-week trial. Summary scores and speech features of individual trials are illustrated for each participant under Supplementary Material. Vowel and consonant data of the 40 participants are presented in the same order as before. Many participants, especially the lowest ranked ones, show little improvement with time because they already perceive the different features very well. However, others show improvements with time, possibly because of practicing the listening training tasks or due to the weekly testing regime of DiN and phoneme identification to know whether a change in summary score is meaningful, histograms of the differences between consecutive scores were constructed for vowels and consonants separately. These are illustrated in Figure 4. The standard deviation of the distribution can be used as a guideline to make changes to the mapping of the device or to the audiological management of the client. For the current data, changes smaller than 10% may be meaningful, but changes larger than 10% certainly are.

### First Versus Last Measurement

**FIGURE 5:**
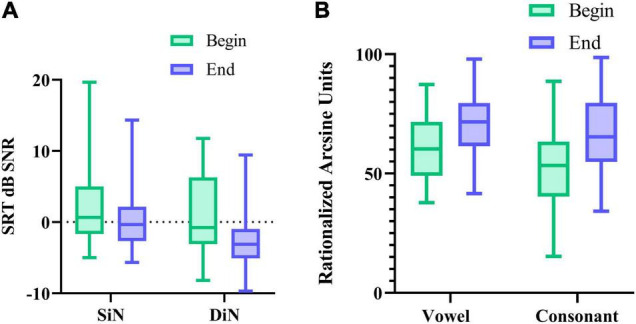
**(A)** median SRT in noise (and IQR) for SiN, DiN, and **(B)** median percentage vowel and consonant identification in quiet (and IQR) at the beginning and end of the 16-week trial.

We also compared potential performance differences between the first and the last measurement. For this, we compared 1 SiN value (streaming mode), the average of 2 DiN scores, and one vowel and one consonant identification score assessed at the beginning of the trials with the same outcomes assessed at the end. Phoneme data were transformed to RAU scores (Studebaker, 1985) for the statistical analyses. Figures 5A,B illustrate the difference in performance between the beginning and end of the period. Wilcoxon signed-rank tests for paired comparisons revealed that speech perception scores were significantly lower (better) after 16 weeks than before for all outcome measures: for SiN *z* = −2.88, *p* = 0.004, for DiN, *z* = −4.7, *p* < 0.0001, for vowel identification in quiet *z* = −4.5, *p* < 0.0001 and consonant identification in quiet *z* = −5.0, *p* < 0.0001. Median and IQR scores are presented in Table 1.

**TABLE 1:** Median SRT in noise and IQR, min and max for SiN in streaming mode, daily settings, and sound-field, only 1 CI (CI-SF) for the first and last session separately (*n* = 40).

	Median	IQR	Min	Max
**First week**				
SiN streaming (dB SNR)	0.7	6.7	−5.0	19.7
SiN daily settings (dB SNR)	0.3	3.8	−3.7	20.0
SiN CI-SF (dB SNR)	1.5	4.9	−3.7	20.0
DiN (dB SNR)	0.02	9.9	−8.8	37.0
Vowel%	61	23.5	37.0	86.0
Consonant%	55	24.6	15.8	88.7
**Last week**				
SiN streaming (dB SNR)	−0.3	4.5	−5.7	14.3
SiN daily settings (dB SNR)	−1.2	4.0	−7.0	9.3
SiN CI-SF (dB SNR)	0.7	5.8	−5.7	20.0
DiN (dB SNR)	−3.2	4.4	−10.0	10.9
Vowel%	72.5	17.5	41.0	93.0
Consonant%	66.3	24.6	33.3	93.3

*Median SRT in noise and IQR, min and max for DTT streaming, the first two sessions (n = 80) or the last two sessions (n = 80) separately. Median vowel percentage (and IQR) of the first vowel identification task and the first consonant identification task (week 1 and 2) as well as for the last two weeks of the trials (week 15 and 16).*

For the sake of comparison, Table 1 also lists the median SiN thresholds for speech stimuli presented to only the CI (CI-SF) and in the daily settings condition (with CI and HA if applicable). These three different SiN outcomes do not differ statistically from each other at the beginning of the trial. However, at the end of the trial, the non-parametric Friedman test of differences among repeated measures rendered a Chi-square value of 12.1, which was significant (*p* < 0.005). A Wilcoxon signed-rank tests subsequently showed that the SiN “daily settings” was significantly better than the SiN in streaming mode (*x* = −2.574, *p* = 0.010) and SiN CI-SF (*X* = −3.968, *p* < 0.0001).

## Discussion

### Sentence and Digits Understanding in Noise

Understanding speech in noise is the most common complaint of persons with hearing impairment, and several indices can be used to document listening difficulties and guide hearing rehabilitation. The present study reports three different indices for speech perception in the same 40 CI users, of which two are administered at home. Where possible, we present individual results instead of group mean average to better understand individual differences in speech recognition outcomes (which, in turn, enables personalized rehabilitation).

SiN performance of contemporary CI users is excellent in the current study, even when the sentences are only streamed to the implanted side. Candidacy criteria for cochlear implantation have changed considerably over the past years, and several CI users have residual hearing (Snel-Bongers et al., 2018). Nevertheless, variability in performance is large, and to understand the source of variability, it is important to look at individual performance and do this from different perspectives. Word and sentence identification remain important measures to evaluate an intervention (e.g., Zhang and Coelho, 2018; Kelsall et al., 2021). While open-set word and sentence understanding lack full external generalizability to speech perception in daily life, they are most closely related to capturing some of the real-world listening difficulties. These measures involve phonological, lexical, grammatical skills, and semantic/contextual knowledge (Heald and Nusbaum, 2014), especially when administered using an open-set response format (Clopper et al., 2006). In an open-set task, listeners compare the stimulus to all possible candidate words in lexical memory.

In contrast, in closed set tests, the listeners need to make only a limited number of comparisons among the response alternatives. An advantage of SiN above word identification is the steeper slope of the performance intensity function of the former. The slope measures how rapidly performance changes with a change in level or signal to noise ratio (Leek, 2001).

However, SiN cannot be assessed too often (due to learning and limited test materials), while DiN can be used repeatedly and without a clinician. The high correlation between DiN and SiN is in line with the results of Smits et al. (2013) for persons with normal hearing and Kaandorp et al. (2015), Kaandorp et al., 2016, [Bibr B32][Bibr B8], and Zhang et al. (2019) for persons with cochlear implants, thereby indicating that the two measures share some common mechanisms. The difference between the two is in the order of 4 dB SNR in the current study. DiN may even be more sensitive than SiN to capture changes in auditory performance, which can be done for each ear separately. The large dataset of the current study did reveal individual differences in performance which do not necessarily change with time. For some participants, especially those struggling most with SiN, performance varied substantially. Here, measurement error of subsequent trials can be used as a guideline for potential changes in performance.

### Phoneme Identification in Quiet

Phoneme identification in quiet sheds additional light on variability in speech perception. While sentence and word recognition in quiet often yield ceiling scores, phoneme scores provide specific information on the perception of speech cues in the absence of context cues. Phoneme perception has a long history in research (Miller and Nicely, 1955) but is not often used as a standard metric in the clinic. At least two arguments plead in favor of incorporating phoneme identification in clinical care. First, it is essential to know how vowel and consonant identification in quiet relate to performance on tests in noise. Our study shows that vowel and consonant recognition in quiet contribute (uniquely) to SiN (and to DiN) and yields additional information on the audibility and discriminability of speech cues. At the clinic, phoneme recognition is often assessed via meaningful words (phoneme score of a word recognition test). However, nonsense syllables are preferred over meaningful words, because context can affect the recognition of phoneme scores in the latter (Donaldson and Kreft, 2006). With a nonsense syllable task, each phoneme can be presented an equal number of times. The task takes only a few minutes and can easily be done remotely. In the future, phoneme perception in noise will also be considered.

Second, phoneme identification can also be used as a diagnostic tool. While percentage correct is a summary score of phoneme perception, the information transmission analyses reveal which spectral and temporal cues are most challenging for the recipient. This information can help optimize the mapping and provide targeted rehabilitation. For instance, a low score for duration discrimination in vowels could guide the clinician to provide tasks to improve discriminability between short and long vowels. A low score on “frication” or “voicing” could guide the clinician to optimize the mapping of high- and low-frequency cues, respectively. The value of this metric was recognized several years ago (van Wieringen and Wouters, 2000) but seemed cumbersome to implement in clinical care. With novel, cost-effective technologies, the benefit of assessing phoneme perception at regular intervals can be reconsidered. Note that sufficient data scores are required to draw conclusions from the information transmission analyses. The maximum-likelihood estimate for information transfer is biased to overestimate its true value when the number of stimulus presentations is small (Sagi and Svirsky, 2008).

### Procedural Learning

Learning either the content or the procedure of a test could improve performance when presented repeatedly. During the 16-weeks, the participants also practiced training modules (Magits et al., under revision). Comparison of SiN pre- versus post-training showed a significant improvement in speech understanding in noise for both the personalized LUISTER and the non-personalized listening training programs. Since the same sentences were never presented twice to the same participant, the observed differences are more likely to result from practicing than repeated testing. However, it is difficult to determine whether the observed improvements for DiN and phoneme identification result from the content of the listening training (perceptual learning) or procedural learning (repeated listening to a task). All perceptual experiments involve some procedural learning, such as getting acquainted with a voice, the characteristics of the speech material, etc., (Nygaard and Pisoni, 1998; Yund and Woods, 2010). A procedural learning effect is larger for a closed-set than for open-set response format, but Smits et al. (2013) report that procedural learning with DiN is accomplished after 1 trial with normal-hearing persons. Nevertheless, de Graaff et al. (2019) report that DiN data of CI users reveal improvements in speech recognition over time, without a clear relation to fitting appointments with an audiologist. These improvements could result from procedural learning or improved perception of speech perception in general.

### Remote Care

Rehabilitation following cochlear implantation is demanding and requires several visits to the clinic to fine-tune the device. With the growing number of clients, improved technology, and public health concerns surrounding the COVID-19 pandemic, remote testing has sparked a lot of interest to complement care at the clinic. The shared responsibility between professional and client may also empower clients to take action if needed. Home-based testing has the potential to change and improve the hearing care pathway. It would not only lead to a reduction in the required number of visits and thus reduction in cost− and time savings for both clinics and patients, it would even improve the quality and richness of data obtained during audiological rehabilitation. The importance of speech in noise testing cannot be overestimated, but note that it entails more than the perception of the auditory signal in noise which can be captured with a DiN task. When the acoustical signal is difficult to perceive, as in noisy conditions, speech understanding places more demands on linguistic knowledge and executive functioning (Mattys et al., 2012; Moberly et al., 2019; Zhan et al., 2020). Remote monitoring of speech-in-noise performance possibly makes room to assess neurocognitive abilities that differentially explain speech in noise performance, which may lead to a personalized holistic management of hearing impairment.

For remote testing to be successful, the obtained data should be clinically valid and accurate, and clients should feel confident handling the device. In our study, all participants felt comfortable doing tests remotely because the professional had provided sufficient information prior to the trial and was online available to address any concerns or technical problems. Data collection with wireless streaming was reliable as repeated testing yielded similar results in the same CI user.

During the COVID-19 pandemic, face-to-face care was brought to a halt, and interest in tele-audiology surged out of necessity. While a recent survey reports that audiologists are generally positive about teleaudiology, infrastructure and training should not be underestimated, and hybrid care remains necessary (Saunders and Roughley, 2021). Also, more research is needed to examine reimbursement and cost-effectiveness of remote services (Bush et al., 2016). Such factors may represent a barrier to the practical delivery of telemedicine services, and these topics represent areas for further research. Technical advances in connectivity now allow for wireless streaming capabilities for current CI systems. Wireless streaming provides good quality audio and is less susceptible to noise or signal processing introduced by the connection cable. Only one calibration is needed for a given digital communication set. In the current study, only the implanted side was assessed at home, but stereo streaming is possible. It remains important to evaluate the whole hearing pathway in the sound field too.

## Conclusion

Speech perception assessment in the same forty CI users showed that DiN assessed at home is a powerful alternative to SiN in the clinic to monitor performance at regular intervals and detect changes in auditory performance. Phoneme identification in quiet also explains a significant part of speech perception in noise and provides additional information on the detectability and discriminability of speech cues. DiN and phoneme identification in quiet can be assessed reliably at home in a limited amount of time. Home-based testing with wireless streaming can be complementary to testing in the clinic. Embracing these technologies could reduce the cost, serve clients who would otherwise not have access to clinical services, and open the door to holistic hearing care.

## Data Availability Statement

The original contributions presented in the study are included in the article/Supplementary Material, further inquiries can be directed to the corresponding author.

## Ethics Statement

The studies involving human participants were reviewed and approved by the Ethics Committee of the University Hospital Leuven. The patients/participants provided their written informed consent to participate in this study.

## Author Contributions

AW, SM, TF, and JW contributed to the conceptualization of this research project. AW wrote the manuscript. JW, TF, and SM provided critical revision and feedback. All authors contributed to the article and approved the submitted version.

## Conflict of Interest

The authors declare that the research was conducted in the absence of any commercial or financial relationships that could be construed as a potential conflict of interest.

## Publisher’s Note

All claims expressed in this article are solely those of the authors and do not necessarily represent those of their affiliated organizations, or those of the publisher, the editors and the reviewers. Any product that may be evaluated in this article, or claim that may be made by its manufacturer, is not guaranteed or endorsed by the publisher.
