# Supplementary material for: Home-Based Speech Perception Monitoring for Clinical Use With Cochlear Implant Users
Source: Front Neurosci. 2021 Nov 30;15:773427. doi: 10.3389/fnins.2021.773427 (PMC8669965; doi:10.3389/fnins.2021.773427)
Supplement: Supplementary file 1 [file Data_Sheet_1.PDF]

Vowel (left) and consonant (right) scores of each participant. Participants are ranked from good to poorer SiN. Figures show the summary score as well as the perception of speech features for each individual trial (indicated by trial number).

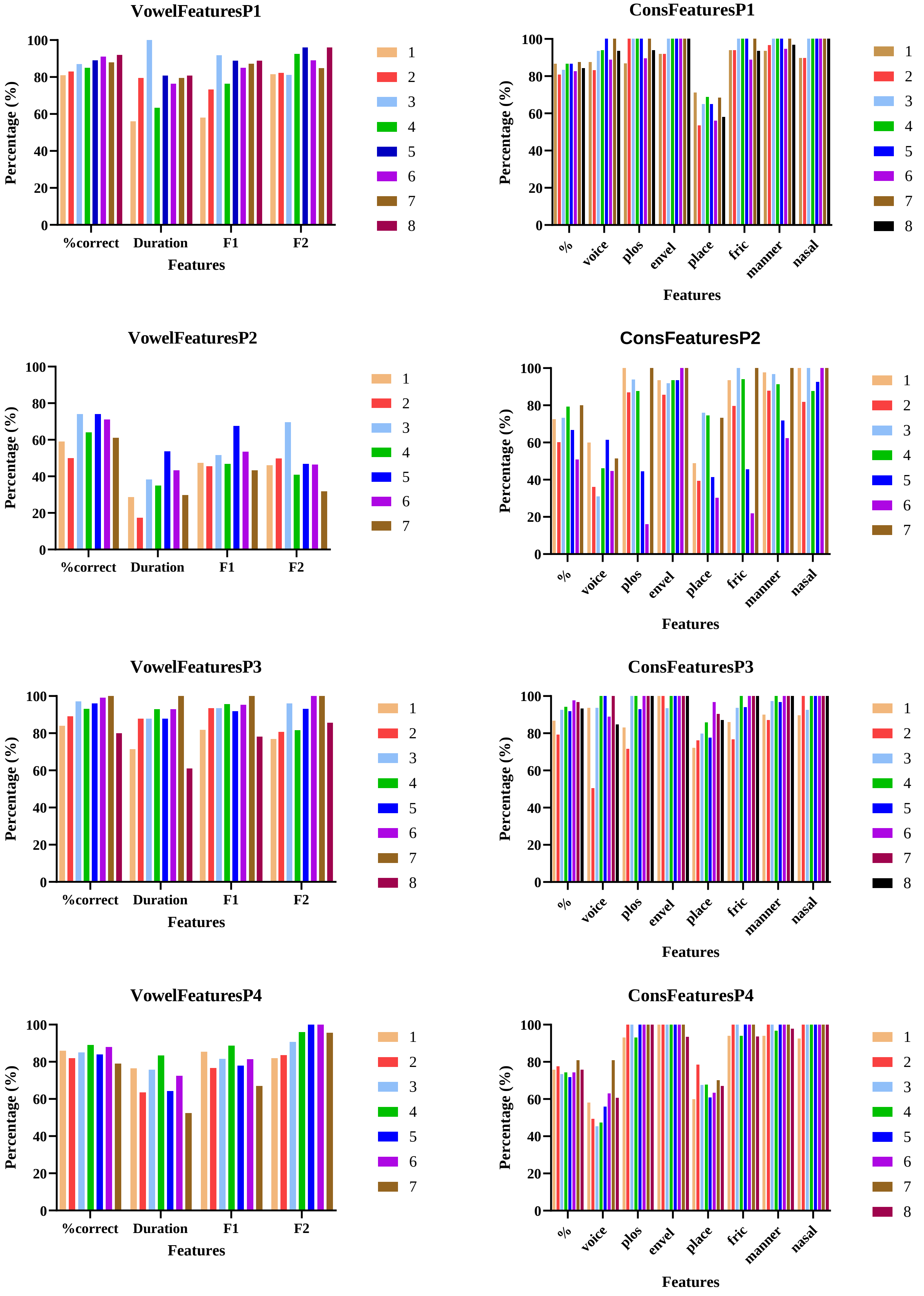

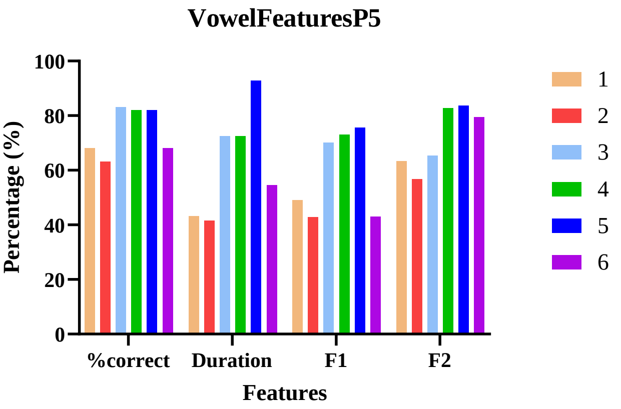
VowelFeaturesP5
Percentage (%)
0
20
40
60
80
100
%correct
Duration
F1
F2
Features
1
2
3
4
5
6

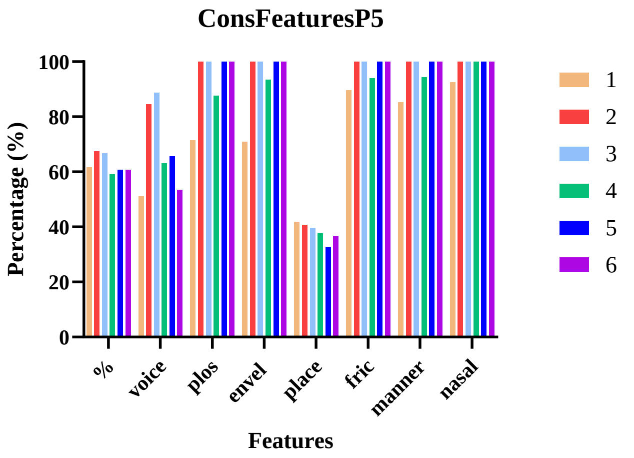
ConsFeaturesP5
Percentage (%)
0
20
40
60
80
100
%
voice
plos
envel
place
fric
manner
nasal
Features
1
2
3
4
5
6

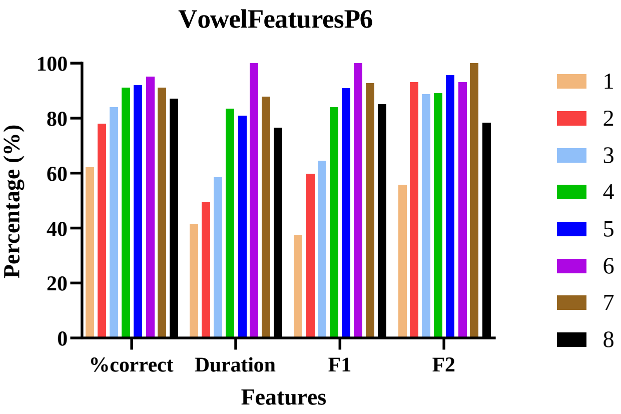
VowelFeaturesP6
Percentage (%)
0
20
40
60
80
100
%correct
Duration
F1
F2
Features
1
2
3
4
5
6
7
8

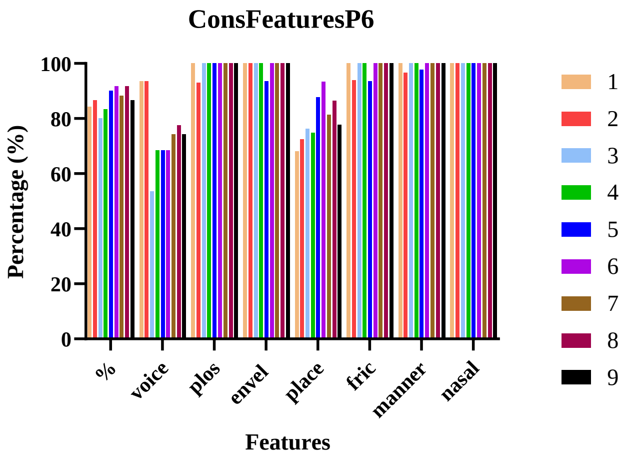
ConsFeaturesP6
Percentage (%)
0
20
40
60
80
100
%
voice
plos
envel
place
fric
manner
nasal
Features
1
2
3
4
5
6
7
8
9

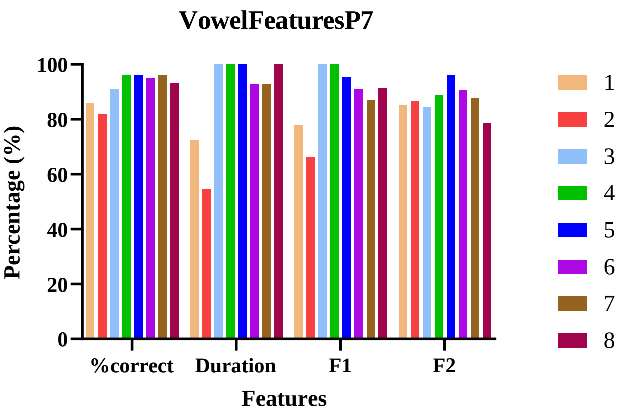
VowelFeaturesP7
Percentage (%)
0
20
40
60
80
100
%correct
Duration
F1
F2
Features
1
2
3
4
5
6
7
8

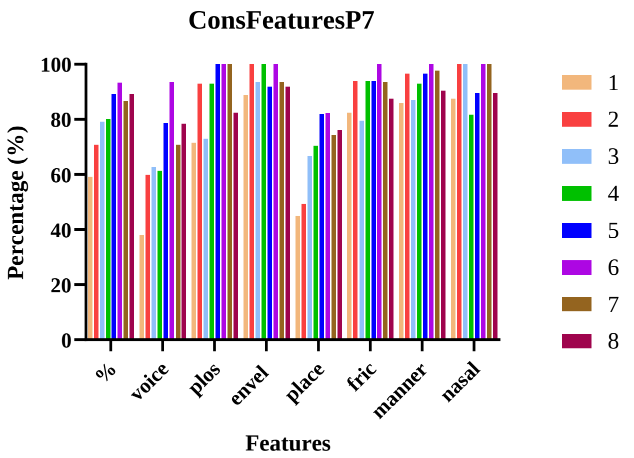
ConsFeaturesP7
Percentage (%)
0
20
40
60
80
100
%
voice
plos
envel
place
fric
manner
nasal
Features
1
2
3
4
5
6
7
8

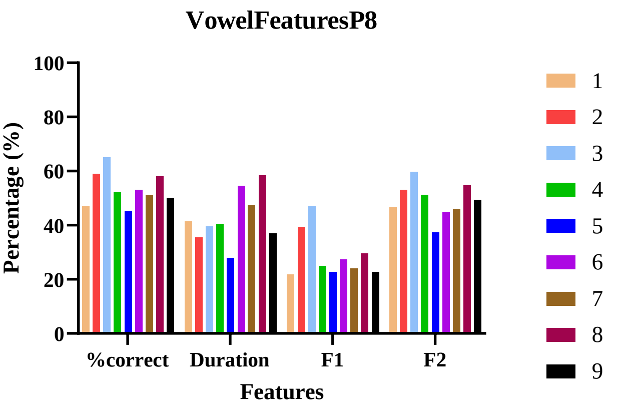
VowelFeaturesP8
Percentage (%)
0
20
40
60
80
100
%correct
Duration
F1
F2
Features
1
2
3
4
5
6
7
8
9

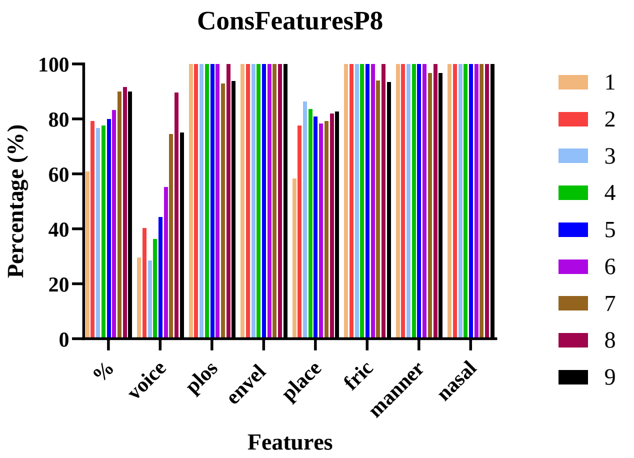
ConsFeaturesP8
Percentage (%)
0
20
40
60
80
100
%
voice
plos
envel
place
fric
manner
nasal
Features
1
2
3
4
5
6
7
8
9

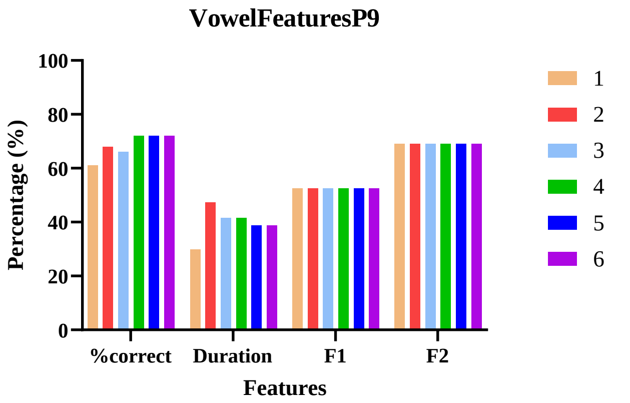
VowelFeaturesP9
Percentage (%)
0
20
40
60
80
100
%correct
Duration
F1
F2
Features
1
2
3
4
5
6

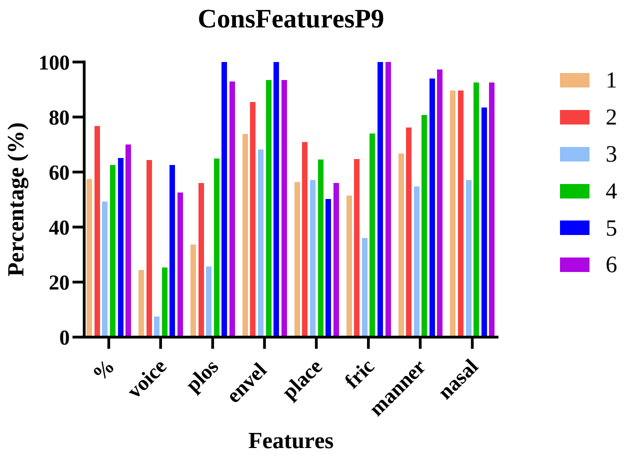
ConsFeaturesP9
Percentage (%)
0
20
40
60
80
100
%
voice
plos
envel
place
fric
manner
nasal
Features
1
2
3
4
5
6

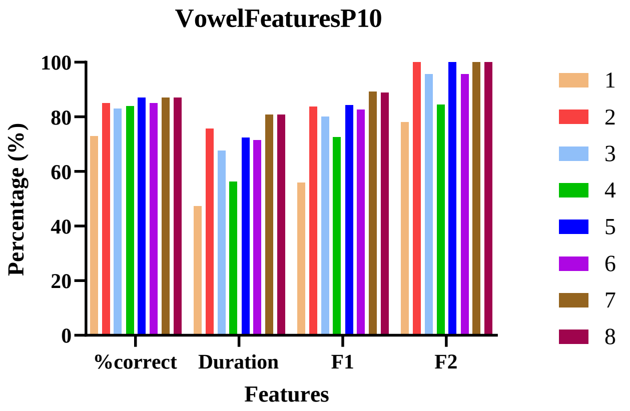
VowelFeaturesP10
Percentage (%)
0
20
40
60
80
100
%correct
Duration
F1
F2
Features
1
2
3
4
5
6
7
8

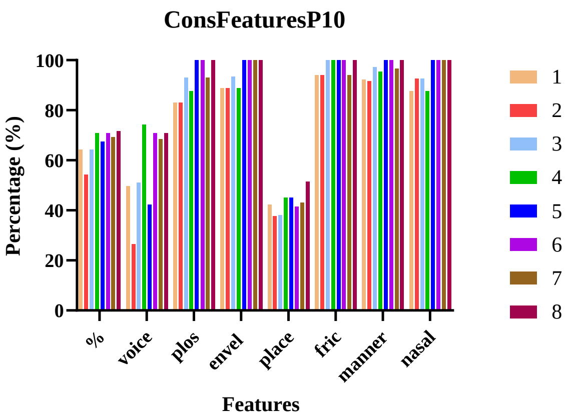
ConsFeaturesP10
Percentage (%)
0
20
40
60
80
100
%
voice
plos
envel
place
fric
manner
nasal
Features
1
2
3
4
5
6
7
8

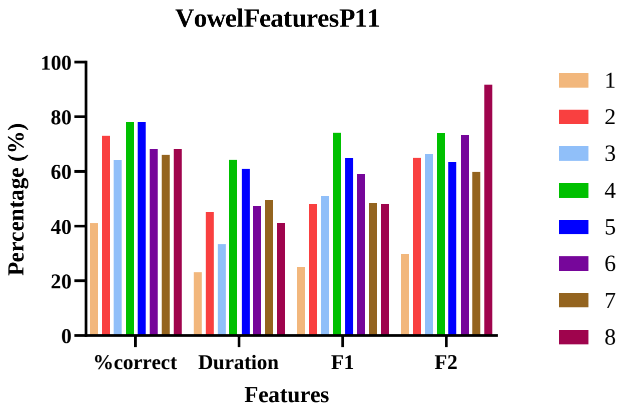
VowelFeaturesP11
Percentage (%)
0
20
40
60
80
100
%correct
Duration
F1
F2
Features
1
2
3
4
5
6
7
8

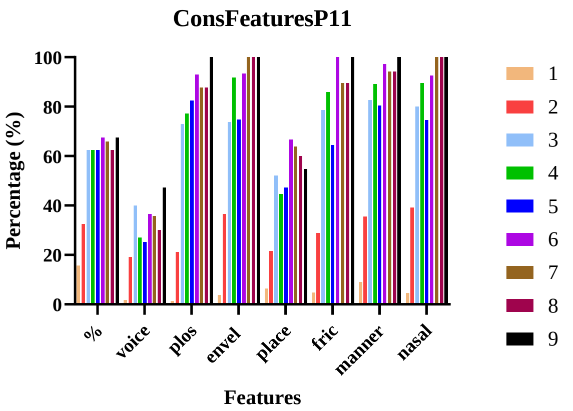
ConsFeaturesP11
Percentage (%)
0
20
40
60
80
100
%
voice
plos
envel
place
fric
manner
nasal
Features
1
2
3
4
5
6
7
8
9

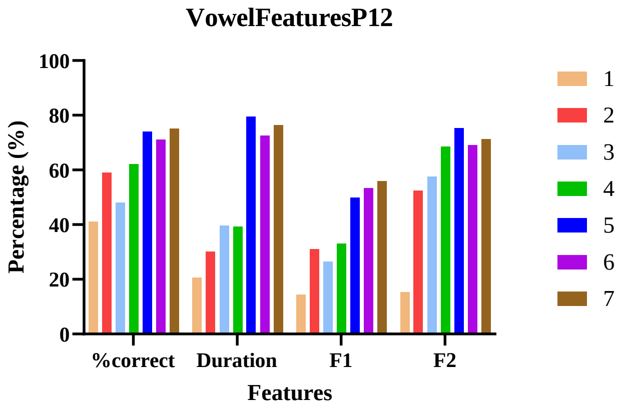
VowelFeaturesP12
Percentage (%)
0
20
40
60
80
100
%correct
Duration
F1
F2
Features
1
2
3
4
5
6
7

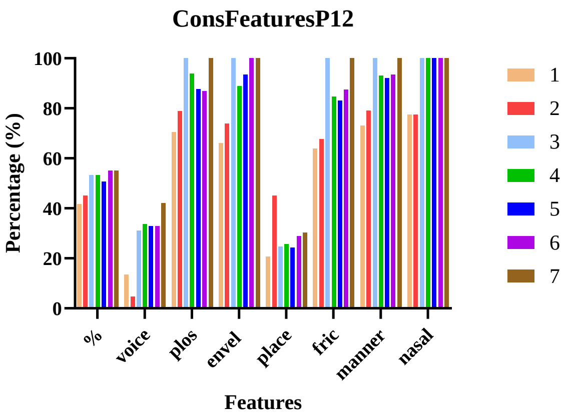
ConsFeaturesP12
Percentage (%)
0
20
40
60
80
100
%
voice
plos
envel
place
fric
manner
nasal
Features
1
2
3
4
5
6
7

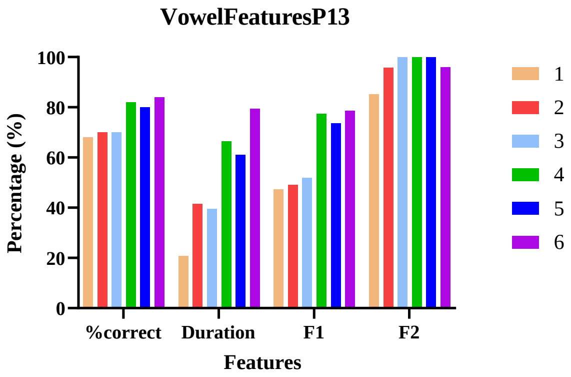
VowelFeaturesP13
Percentage (%)
0
20
40
60
80
100
%correct
Duration
F1
F2
Features
1
2
3
4
5
6

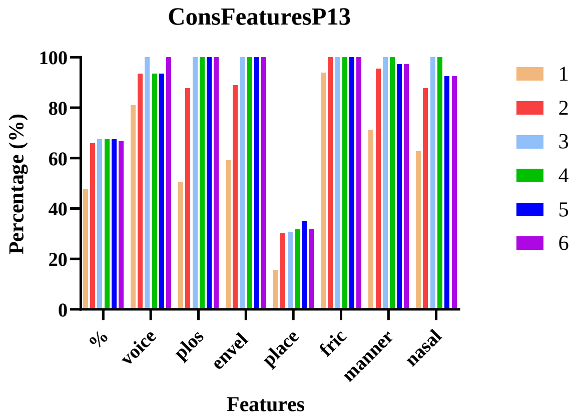
ConsFeaturesP13
Percentage (%)
0
20
40
60
80
100
%
voice
plos
envel
place
fric
manner
nasal
Features
1
2
3
4
5
6

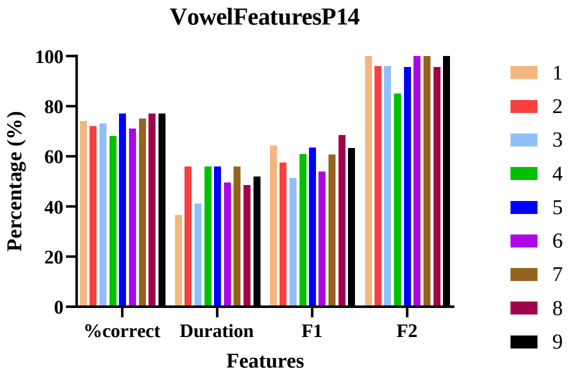
VowelFeaturesP14
Percentage (%)
0
20
40
60
80
100
%correct
Duration
F1
F2
Features
1
2
3
4
5
6
7
8
9

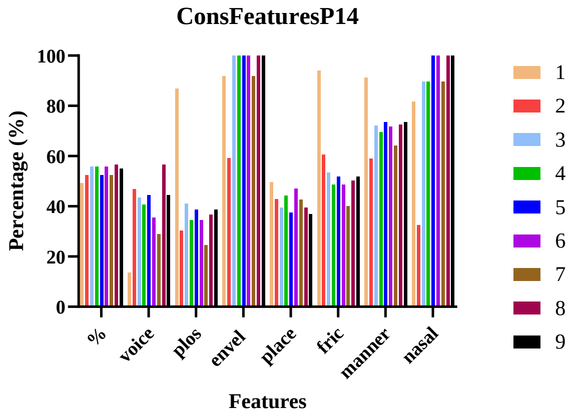
ConsFeaturesP14
Percentage (%)
0
20
40
60
80
100
%
voice
plos
envel
place
fric
manner
nasal
Features
1
2
3
4
5
6
7
8
9

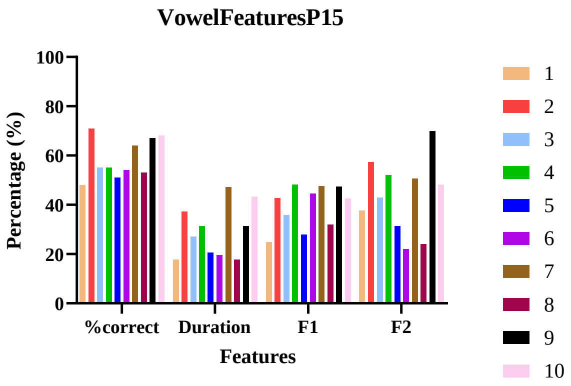
VowelFeaturesP15
Percentage (%)
0
20
40
60
80
100
%correct
Duration
F1
F2
Features
1
2
3
4
5
6
7
8
9
10

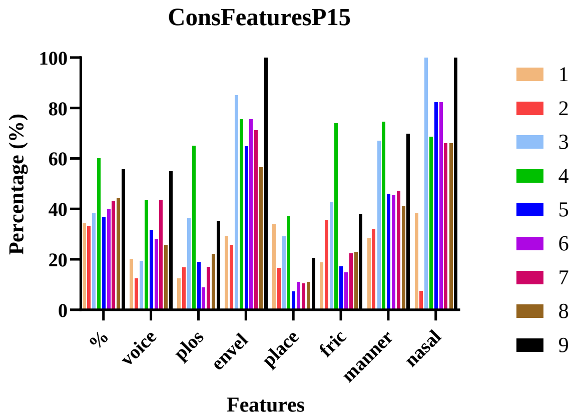
ConsFeaturesP15
Percentage (%)
0
20
40
60
80
100
%
voice
plos
envel
place
fric
manner
nasal
Features
1
2
3
4
5
6
7
8
9

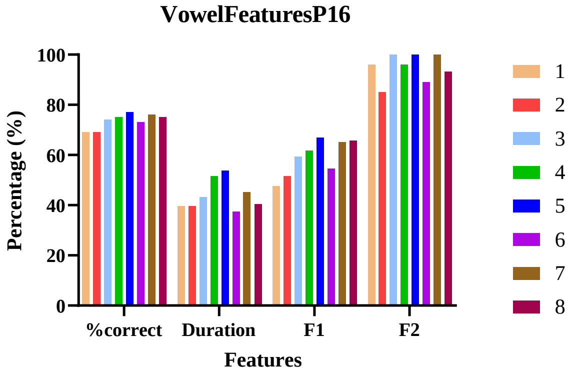
VowelFeaturesP16
Percentage (%)
0
20
40
60
80
100
%correct
Duration
F1
F2
Features
1
2
3
4
5
6
7
8

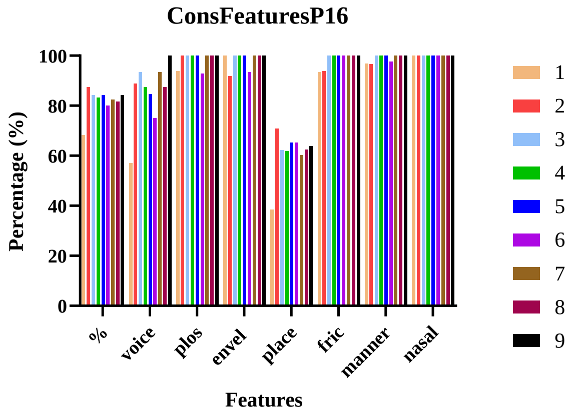
ConsFeaturesP16
Percentage (%)
0
20
40
60
80
100
%
voice
plos
envel
place
fric
manner
nasal
Features
1
2
3
4
5
6
7
8
9

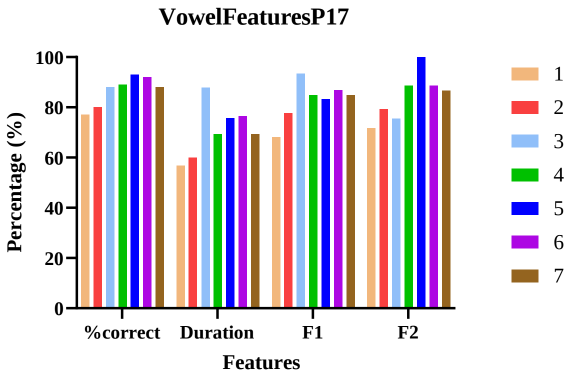
VowelFeaturesP17
Percentage (%)
0
20
40
60
80
100
%correct
Duration
F1
F2
Features
1
2
3
4
5
6
7

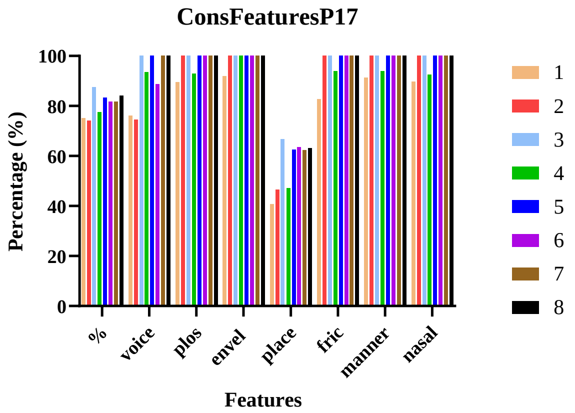
ConsFeaturesP17
Percentage (%)
0
20
40
60
80
100
%
voice
plos
envel
place
fric
manner
nasal
Features
1
2
3
4
5
6
7
8

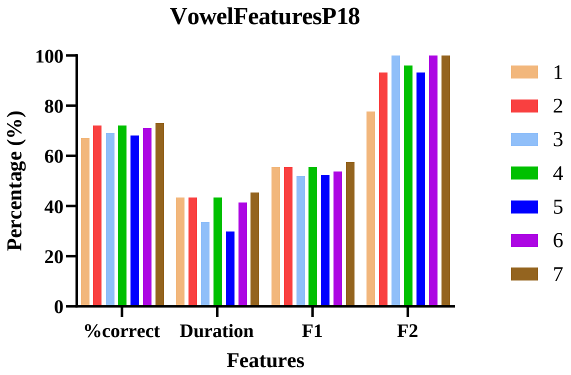
VowelFeaturesP18
Percentage (%)
0
20
40
60
80
100
%correct
Duration
F1
F2
Features
1
2
3
4
5
6
7

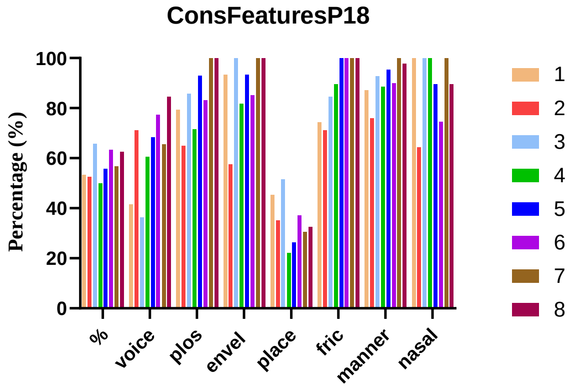
ConsFeaturesP18
Percentage (%)
0
20
40
60
80
100
%
voice
plos
envel
place
fric
manner
nasal
1
2
3
4
5
6
7
8

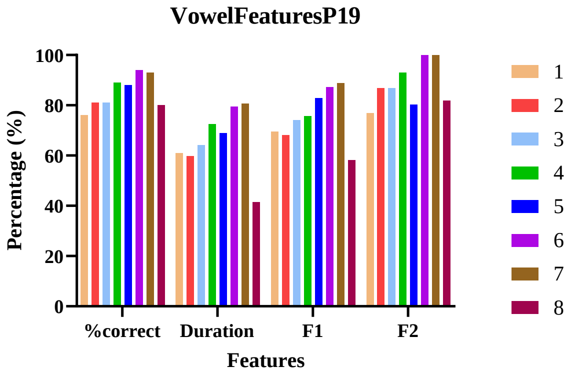
VowelFeaturesP19
Percentage (%)
0
20
40
60
80
100
%correct
Duration
F1
F2
Features
1
2
3
4
5
6
7
8

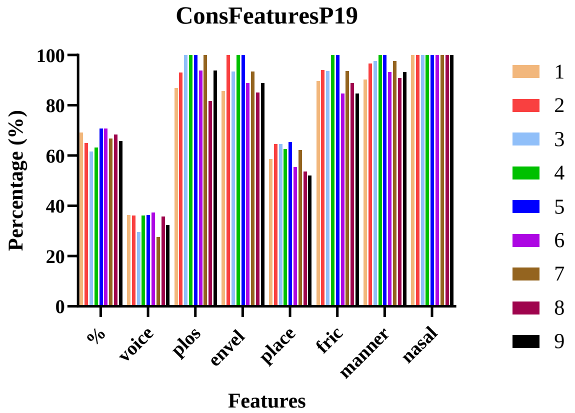
ConsFeaturesP19
Percentage (%)
0
20
40
60
80
100
%
voice
plos
envel
place
fric
manner
nasal
Features
1
2
3
4
5
6
7
8
9

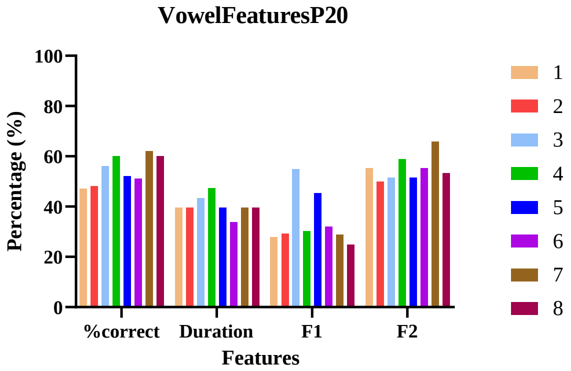
VowelFeaturesP20
Percentage (%)
0
20
40
60
80
100
%correct
Duration
F1
F2
Features
1
2
3
4
5
6
7
8

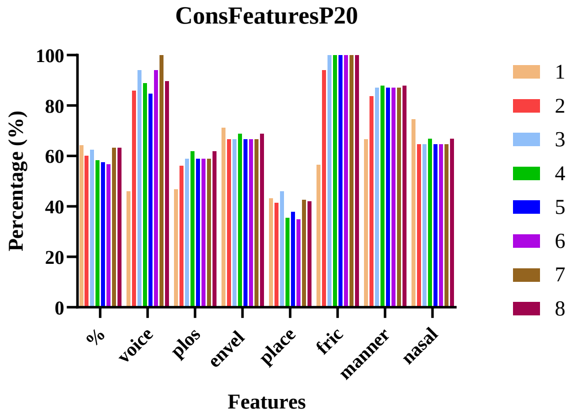
ConsFeaturesP20
Percentage (%)
0
20
40
60
80
100
%
voice
plos
envel
place
fric
manner
nasal
Features
1
2
3
4
5
6
7
8

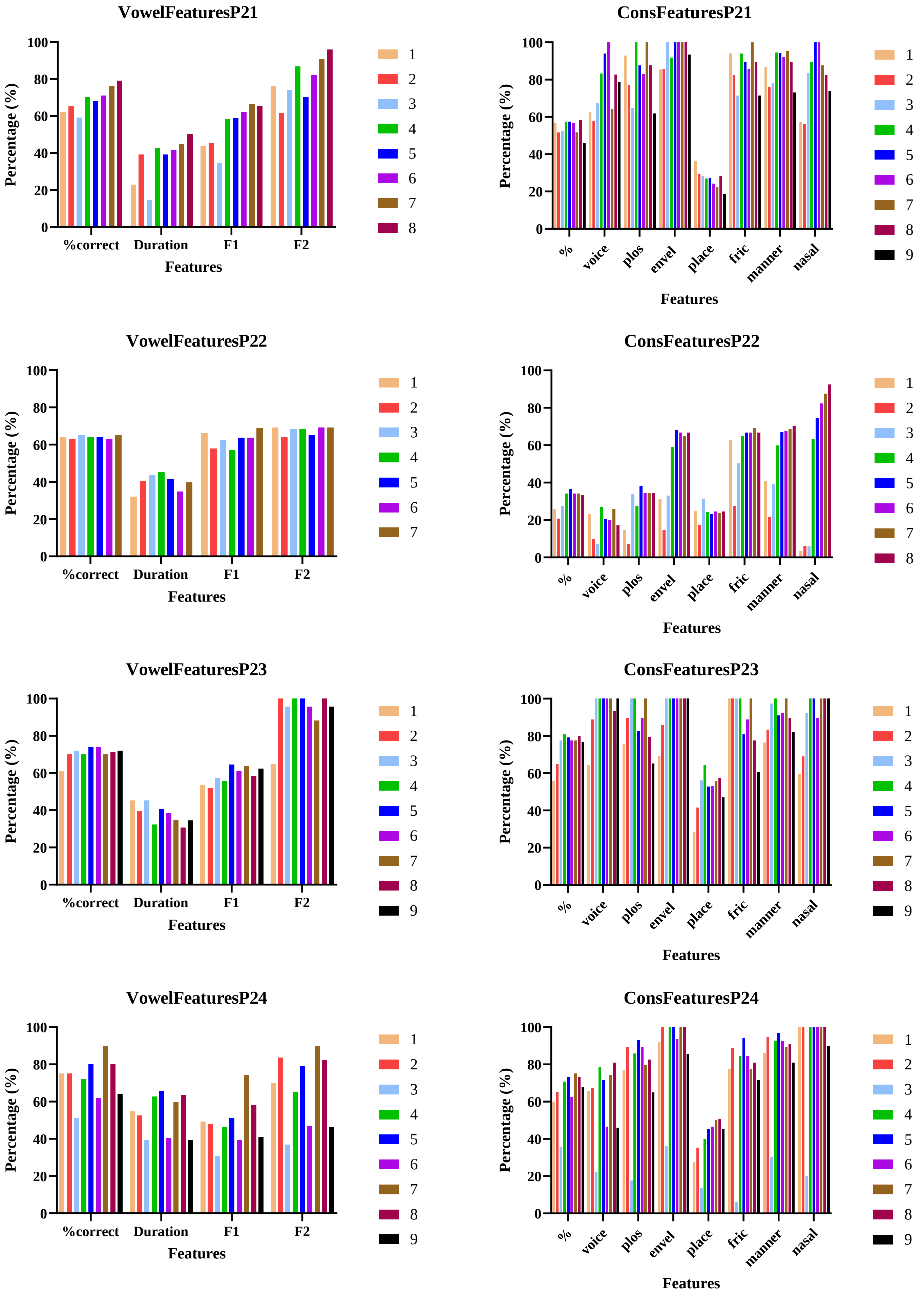
VowelFeaturesP21
ConsFeaturesP21
VowelFeaturesP22
ConsFeaturesP22
VowelFeaturesP23
ConsFeaturesP23
VowelFeaturesP24
ConsFeaturesP24
Percentage (%)
0
20
40
60
80
100
%correct
Duration
F1
F2
%
voice
plos
envel
place
fric
manner
nasal
Features
1
2
3
4
5
6
7
8
9

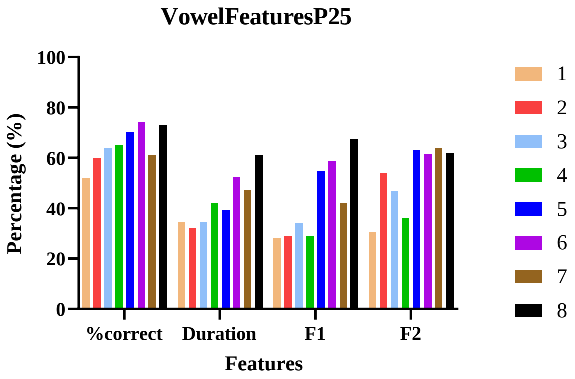
VowelFeaturesP25
Percentage (%)
0
20
40
60
80
100
%correct
Duration
F1
F2
Features
1
2
3
4
5
6
7
8

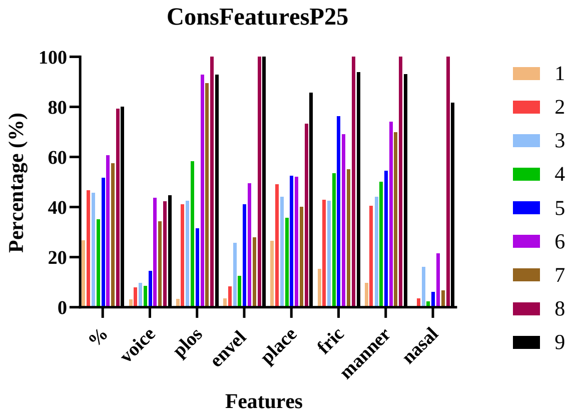
ConsFeaturesP25
Percentage (%)
0
20
40
60
80
100
%
voice
plos
envel
place
fric
manner
nasal
Features
1
2
3
4
5
6
7
8
9

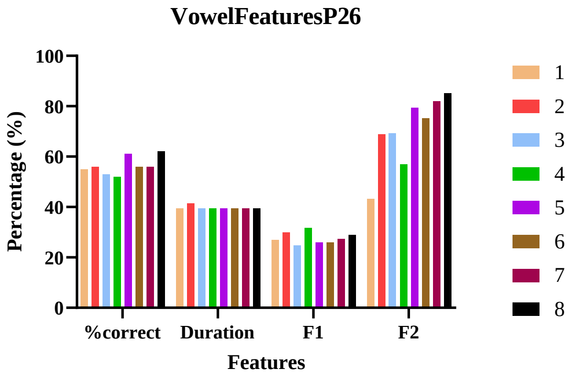
VowelFeaturesP26
Percentage (%)
0
20
40
60
80
100
%correct
Duration
F1
F2
Features
1
2
3
4
5
6
7
8

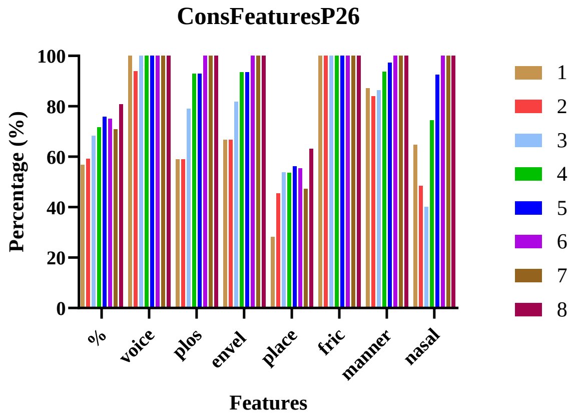
ConsFeaturesP26
Percentage (%)
0
20
40
60
80
100
%
voice
plos
envel
place
fric
manner
nasal
Features
1
2
3
4
5
6
7
8

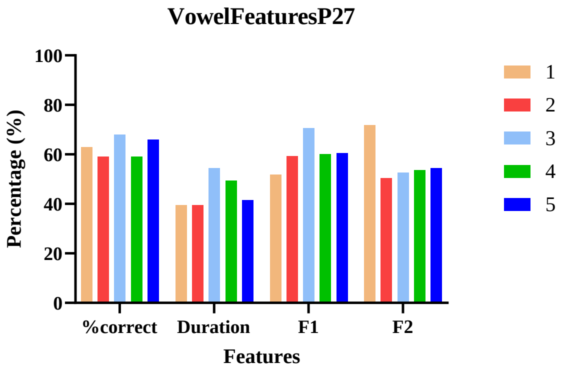
VowelFeaturesP27
Percentage (%)
0
20
40
60
80
100
%correct
Duration
F1
F2
Features
1
2
3
4
5

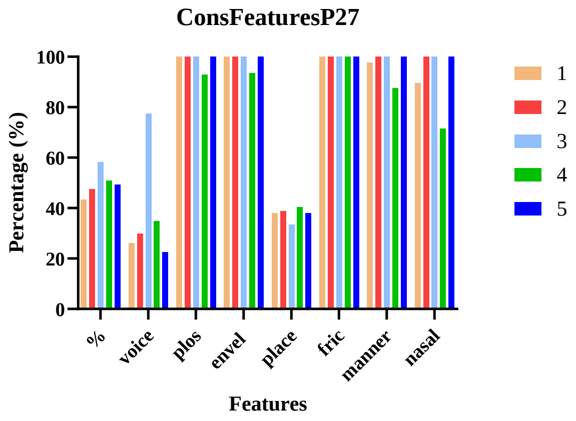
ConsFeaturesP27
Percentage (%)
0
20
40
60
80
100
%
voice
plos
envel
place
fric
manner
nasal
Features
1
2
3
4
5

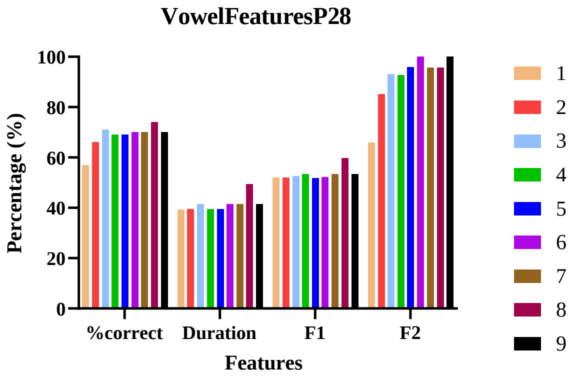
VowelFeaturesP28
Percentage (%)
0
20
40
60
80
100
%correct
Duration
F1
F2
Features
1
2
3
4
5
6
7
8
9

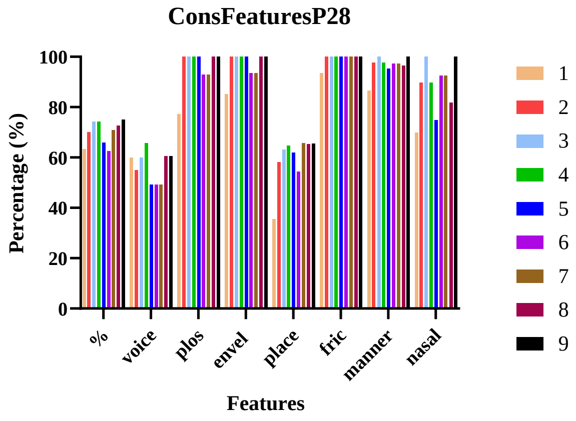
ConsFeaturesP28
Percentage (%)
0
20
40
60
80
100
%
voice
plos
envel
place
fric
manner
nasal
Features
1
2
3
4
5
6
7
8
9

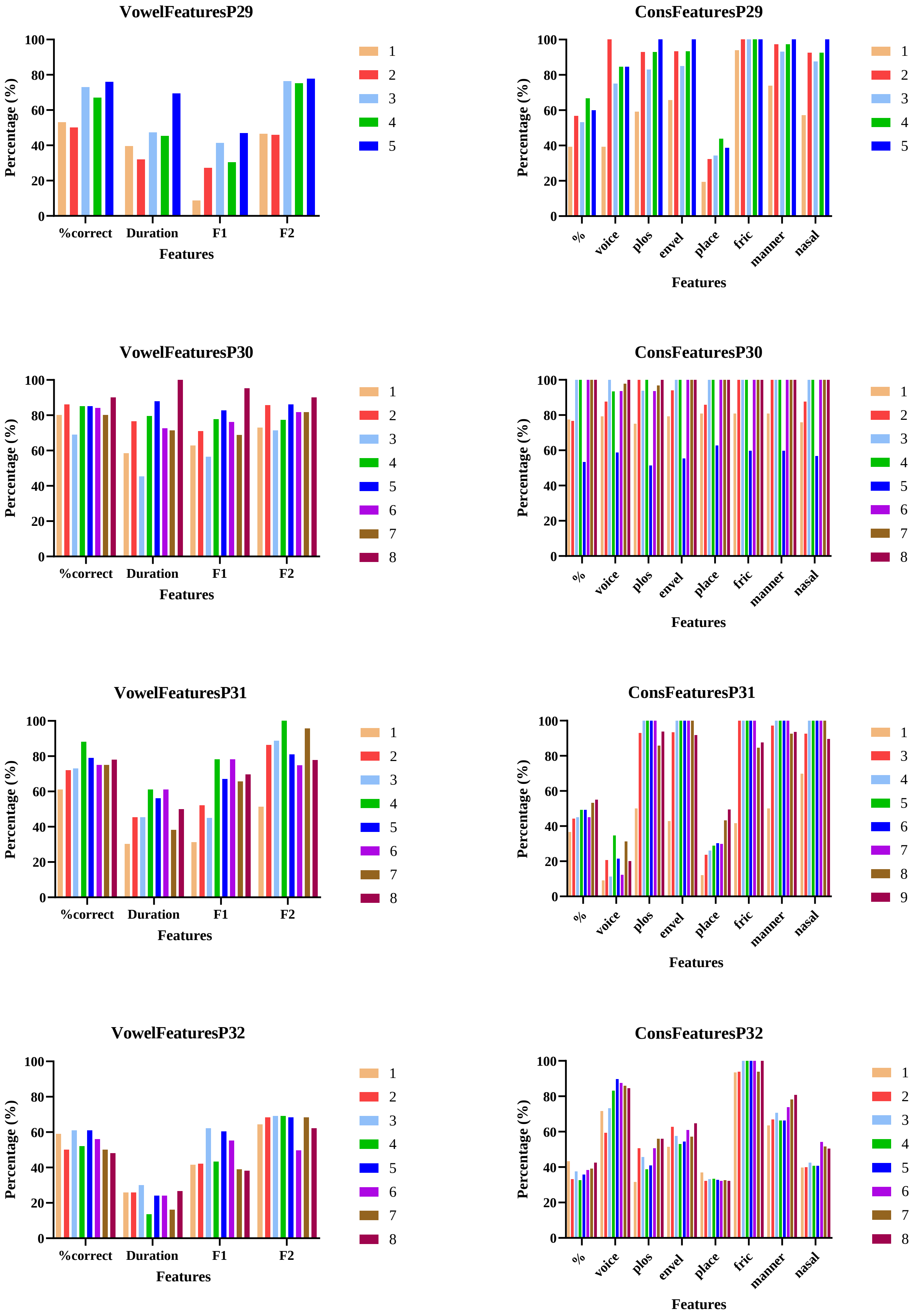
VowelFeaturesP29
ConsFeaturesP29
VowelFeaturesP30
ConsFeaturesP30
VowelFeaturesP31
ConsFeaturesP31
VowelFeaturesP32
ConsFeaturesP32
Percentage (%)
Features
%correct
Duration
F1
F2
%
voice
plos
envel
place
fric
manner
nasal
0
20
40
60
80
100
1
2
3
4
5
6
7
8
9

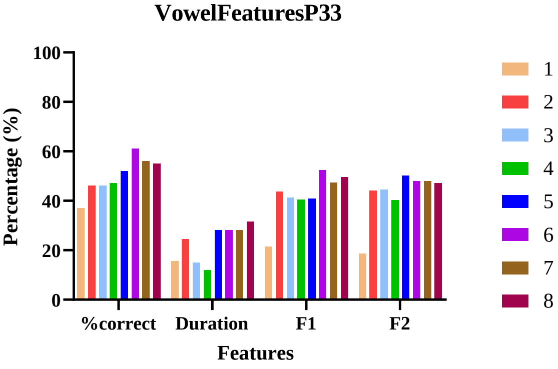
VowelFeaturesP33
Percentage (%)
0
20
40
60
80
100
%correct
Duration
F1
F2
Features
1
2
3
4
5
6
7
8

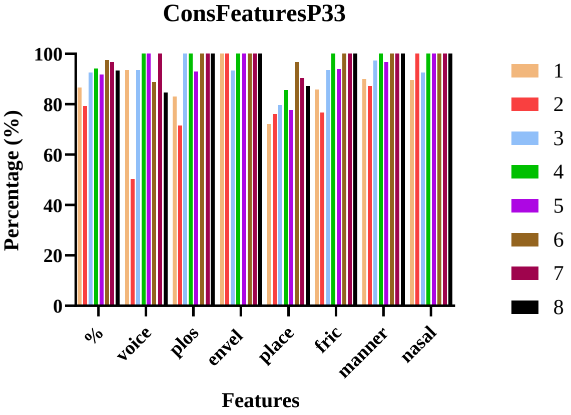
ConsFeaturesP33
Percentage (%)
0
20
40
60
80
100
%
voice
plos
envel
place
fric
manner
nasal
Features
1
2
3
4
5
6
7
8

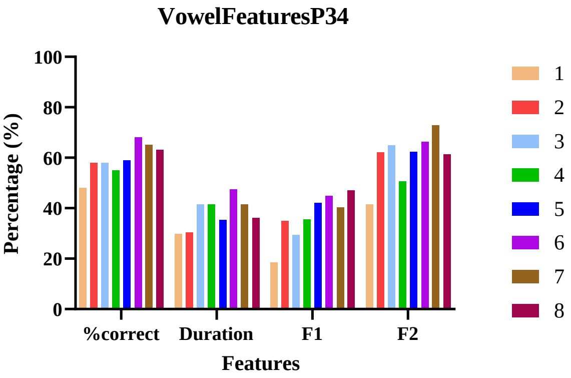
VowelFeaturesP34
Percentage (%)
0
20
40
60
80
100
%correct
Duration
F1
F2
Features
1
2
3
4
5
6
7
8

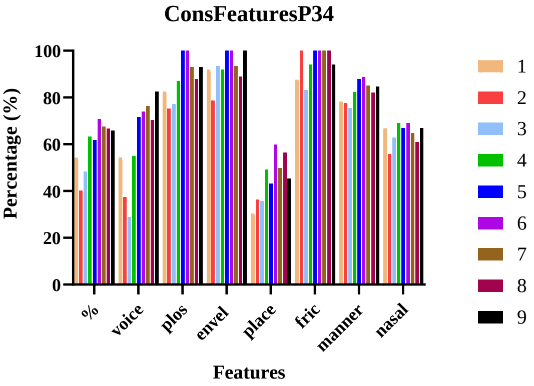
ConsFeaturesP34
Percentage (%)
0
20
40
60
80
100
%
voice
plos
envel
place
fric
manner
nasal
Features
1
2
3
4
5
6
7
8
9

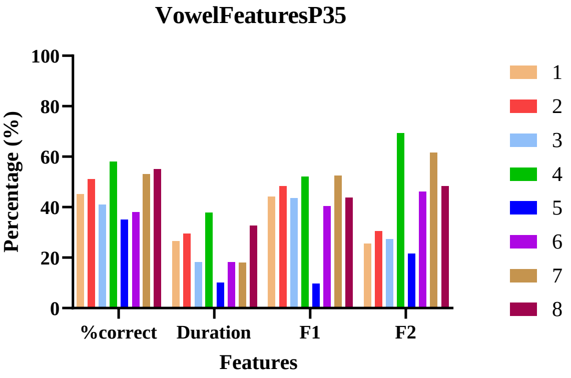
VowelFeaturesP35
Percentage (%)
0
20
40
60
80
100
%correct
Duration
F1
F2
Features
1
2
3
4
5
6
7
8

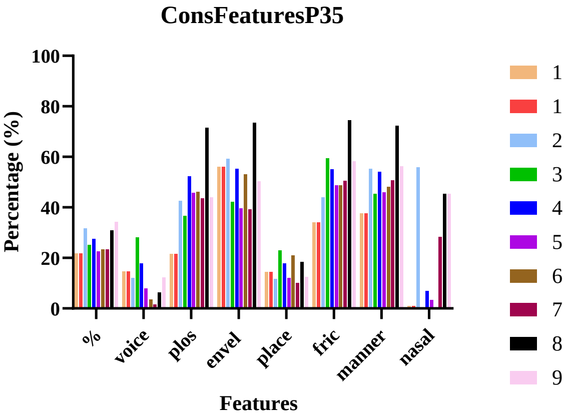
ConsFeaturesP35
Percentage (%)
0
20
40
60
80
100
%
voice
plos
envel
place
fric
manner
nasal
Features
1
1
2
3
4
5
6
7
8
9

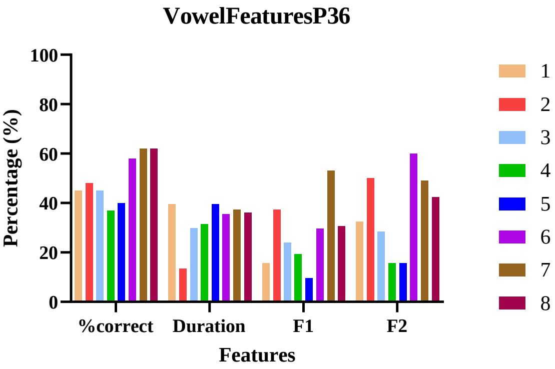
VowelFeaturesP36
Percentage (%)
0
20
40
60
80
100
%correct
Duration
F1
F2
Features
1
2
3
4
5
6
7
8

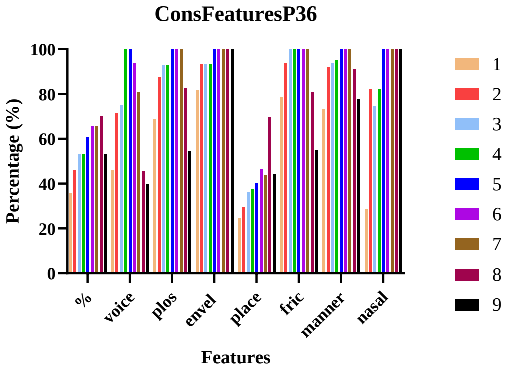
ConsFeaturesP36
Percentage (%)
0
20
40
60
80
100
%
voice
plos
envel
place
fric
manner
nasal
Features
1
2
3
4
5
6
7
8
9

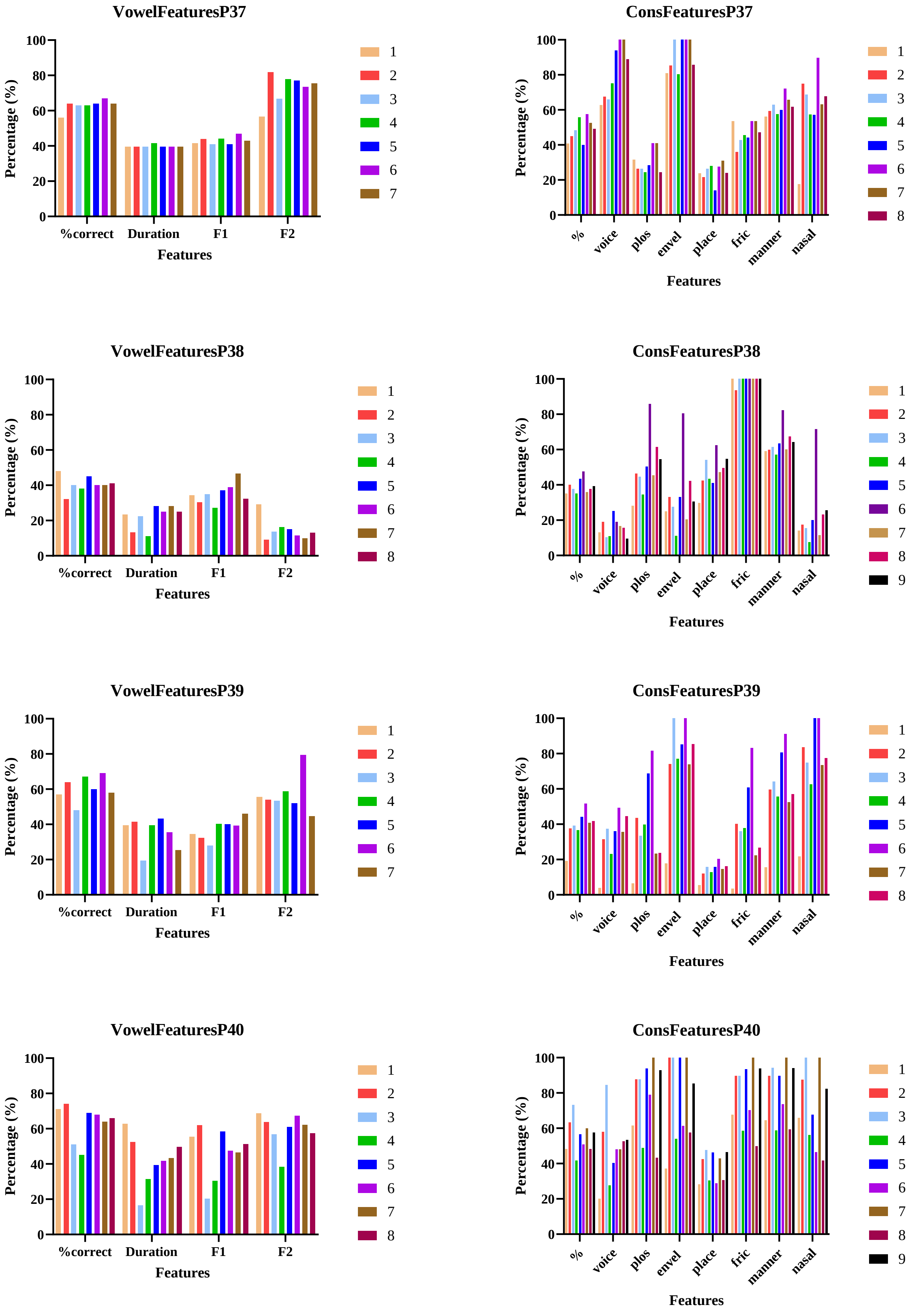
VowelFeaturesP37
ConsFeaturesP37
VowelFeaturesP38
ConsFeaturesP38
VowelFeaturesP39
ConsFeaturesP39
VowelFeaturesP40
ConsFeaturesP40
Percentage (%)
Features
%correct
Duration
F1
F2
%
voice
plos
envel
place
fric
manner
nasal
